# Assessment of Efficacy and Safety of Lipid-Lowering Treatment and Its Importance in Risk Assessment and Prevention in a Hungarian Myositis Cohort

**DOI:** 10.3390/jcm14103404

**Published:** 2025-05-13

**Authors:** Dorottya Szinay, Katalin Szabó, Henrik Molnár, Tibor Béldi, Viktor Bencs, Hajnalka Lőrincz, Mariann Harangi, Zoltán Griger, Melinda Nagy-Vincze

**Affiliations:** 1 Division of Immunology, Department of Internal Medicine, Faculty of Medicine, University of Debrecen, 4032 Debrecen, Hungary; szinay.dorottya@med.unideb.hu (D.S.); szabo.katalin15@gmail.com (K.S.); molnar.henrik1998@gmail.com (H.M.); beldi.tibor@med.unideb.hu (T.B.); griger.zoltan@med.unideb.hu (Z.G.); 2 Gyula Petrányi Doctoral School of Allergy and Clinical Immunology, University of Debrecen, 4032 Debrecen, Hungary; 3 Department of Neurology, Faculty of Medicine, University of Debrecen, 4032 Debrecen, Hungary; bencs.viktor@med.unideb.hu; 4 Doctoral School of Neuroscience, University of Debrecen, 4032 Debrecen, Hungary; 5 Division of Metabolism, Department of Internal Medicine, Faculty of Medicine, University of Debrecen, 4032 Debrecen, Hungary; lorincz_hajnalka@belklinika.com (H.L.); harangi@belklinika.com (M.H.); 6 Faculty of Health Sciences, Institute of Health Studies, University of Debrecen, 4032 Debrecen, Hungary

**Keywords:** idiopathic inflammatory myopathies, cardiovascular disease risk, statin, efficacy

## Abstract

**Background:** Idiopathic inflammatory myopathies (IIMs), also known as myositis, are systemic autoimmune diseases characterized by chronic inflammation affecting the skin, muscles, and internal organs. Besides traditional risk factors and immune-mediated myocarditis, continuous activity of the immune system increases cardiovascular disease (CVD) risk, meaning that cardiovascular events are the leading causes of mortality in IIM patients. Statins are the most widely used lipid-lowering therapies, which reduce cardiovascular risk, but the fear of adverse muscular events inhibits the frequency of use. **Methods:** Our aim was to assess the CVD risk in a myositis cohort using the SCORE2 prediction system, carotid artery Doppler ultrasound measurement, and biomarkers; recommend individual lipid-lowering treatment; and follow the efficacy and adverse events of therapy in a 6-month treatment period. **Results:** The study population (80 IIM patients) was a middle-aged, female-dominant myositis cohort with an average disease duration of 9 years and low median global disease activity. Based on the SCORE2 evaluation, 78.8% of patients had medium/high CVD risk, while 73.13% had asymptomatic carotid plaque. After 6 months of adequate lipid-lowering therapy, 37.5% of patients reached a lower CVD risk category, the biomarker levels of atherosclerosis significantly decreased, and no progression in carotid plaques was detected. None of the patients reported an adverse muscular event or IIM relapse. **Conclusions:** Our findings proved that the CVD risk of patients with myositis is high, but carefully applied lipid-lowering treatment is the key to effective risk reduction. Risk stratification and the recommendation of preventive treatment are the responsibility of the treating physician.

## 1. Introduction

Idiopathic inflammatory myopathies (IIMs) represent a heterogeneous group of autoimmune diseases characterized by proximal muscle weakness, elevated muscle enzyme levels, the presence of various myositis-specific and myositis-associated autoantibodies, and distinct electromyographic abnormalities. The primary subtypes comprise dermatomyositis ((DM) including amyopathic dermatomyositis), anti-synthetase syndrome (ASS), immune-mediated necrotizing myopathy (IMNM), inclusion body myositis (IBM), polymyositis (PM), and overlap myositis (OM), each distinguished by their clinical, histopathological, and immunological features [1]. The main internal organ involvements are interstitial lung disease, dysphagia, and cardiac manifestations. Obvious cardiac abnormalities—myocarditis, pericarditis, and heart failure—are relatively rare, but incidence data vary from 6 to 75%. Subclinical (ECG) manifestations, on the other hand, are more common [2,3,4,5,6,7,8].

Cardiovascular diseases are currently the leading cause of mortality worldwide [9]. Patients with inflammatory rheumatic diseases, including IIM, exhibit a heightened risk of developing cardiovascular disease when compared to the general population. The higher cardiovascular disease (CVD) risk in patients with rheumatic diseases can be attributed to multiple reasons. Several studies reported a higher prevalence of atherosclerosis compared with the general population [3,10,11,12,13,14]. In addition to traditional risk factors, such as hyperlipidaemia, atherosclerosis, smoking, advanced age, hypertension, positive family history, and obesity, the presence of chronic inflammation plays a key role in CVD pathogenesis. The negative effects of chronic glucocorticoid therapy on metabolism cannot be overlooked [3,11,12,13,14]. This risk is a 2.2–2.3-fold increase in the first 5 years after IIM diagnosis [3,8,12,15,16,17,18,19]. Some studies [20] have reported that this increased CVD risk is mostly affected by traditional risk factors, while others have suggested high risk in IIM patients compared to healthy controls independently of traditional cardiovascular risk [12]. Cohort studies have also reported that IIM patients’ major cardiovascular events (stroke and acute coronary disease) occur with higher incidence compared to the general population [15,16,17,18,19,21,22]. Electrocardiography, echocardiography, cardiac magnetic resonance imaging, the determination of laboratory markers, and risk factor evaluations are widely used for CVD risk stratification [3,8,12,23,24,25]. Several cardiovascular risk calculators are available online to measure the probability of developing CVD, such as the Framingham Risk Score (FRS), Systematic Coronary Risk Evaluation (SCORE), American College of Cardiology/American Heart Association (ACC/AHA) Atherosclerotic Cardiovascular Disease Risk Estimator, and the United Kingdom score, which is called QRISK^®^, all designed for the general population. Recommendations are scarce considering CVD prevention protocols and the frequency of risk assessment in IIM patients.

Hyperlipidaemia is the major risk factor of cardiovascular morbidity and mortality in the general population. Statins are the first-line treatment due to their effectiveness in lowering low-density lipoprotein-cholesterol (LDL-C) and reducing cardiovascular risk. Other medications include ezetimibe, which reduces cholesterol absorption, and proprotein convertase subtilisin/kexin type 9 (PCSK9) inhibitors, which further lower LDL-C levels in high-risk patients. Statins inhibit the enzyme 3-hydroxy-3-methylglutaryl coenzyme-A reductase (HMG-CoA). The most common side effects of statins are statin-associated muscle symptoms, which include various types of mechanisms and pathophysiology. It is important to distinguish statin myotoxicity and statin-induced IMNM. While statin myotoxicity usually appears early after statin initiation and resolves with statin cessation, the IMNM appears months to years after statin initiation, does not resolve with statin cessation, and can lead to the presence of the anti-3-hydroxy-3-methylglutaryl coenzyme-A reductase antibody (anti-HMGCR) [13,24,26]. While there has been increasing knowledge about the pathomechanism and outcome of necrotizing myopathy, the fear of statin-induced myopathy has decreased the frequency of use, although its role in CVD risk reduction is obvious [27]. Direct cyto- or myotoxicity of anti-HMGCR antibodies has also been proven [28].

Based on the leading European rheumatology association’s (European Alliance of Associations for Rheumatology—EULAR) recommendations, rheumatologists are responsible for CVD risk assessment and management in collaboration with primary care providers, internists, cardiologists, and other healthcare providers. While several lipid-lowering guidelines are available for the general population, IIM- or RMD-specific guidelines are scarce. International rheumatology guidelines postulate that lipid management should follow recommendations used in the general population [14]. Small case studies have reported severe muscular complications with lipid-lowering therapies in patients with metabolic myopathies [29]. Meanwhile, other well-organized studies proved that statins are well tolerated in myositis patients with careful application [29,30]. In addition to CV benefits, statins have been shown to have other pleiotropic effects, such as the downregulation of multiple inflammatory pathways, which support the argument for further advantages of this lipid-lowering treatment [31]. Thus, to genuinely estimate the risk/benefit ratio of statin use in myositis patients, further cohort analysis is required.

Therefore, our aims were (1) to assess the CVD risk of our patients using the SCORE2 prediction system, carotid artery Doppler ultrasound, intima-media thickness (IMT) measurement, and biomarkers; (2) to recommend individual lipid-lowering treatment (statin or ezetimibe); and (3) to follow the efficacy and adverse events of therapy in a 6-month treatment period. The primary endpoint was the change in CVD risk determined by the SCORE2 method after treatment. The secondary endpoint was to assess the proportion of patients in the cohort who reached a lower SCORE2 category after treatment.

## 2. Materials and Methods

### 2.1. Study Population

Eighty IIM patients participated in this study, who were treated and regularly controlled at the Division of Immunology, Department of Internal Medicine, Faculty of Medicine, University of Debrecen, Debrecen, Hungary. Inclusion criteria were that the patient fulfilled the definition of definite or probable inflammatory myopathy based on the EULAR/ACR classification criteria [32] and gave informed written consent. The laboratory was approved by the National Public Health and Medical Officer Service (approval number: 094025024).

The epidemiology data, general characteristics of IIM disease such as symptoms at disease onset, autoantibody profile, comorbidities, and previous lipid-lowering and immunosuppressive treatments were collected during the screening visit. Myositis-specific and associated antibodies were tested by line blot immunoassay (LIA) using the EUROLINE Autoimmune Inflammatory Myopathies 16Ag kit, which can detect 12 MSA (anti-Mi2A, anti-Mi2B, anti-TIF1γ, anti-MDA5, anti-NXP2, anti-SAE1, anti-SRP, anti-Jo1, anti-PL7, anti-PL12, anti-EJ, and anti-OJ) and 4 MAAs (anti-Ku, anti-PM-Scl100, anti-PM-Scl75, and anti-Ro52) (Euroline Myositis Antigen Profile4, EuroImmun, Lübeck, Germany) according to the manufacturer’s instructions. HMCGR antibodies were detected by indirect immunofluorescence assay and confirmed by in-house ELISA (the cut-off value was ≥40 U/mL).

### 2.2. Sample Collection and Biochemical Measurements

All venous blood samples were drawn after 12 h of fasting. Routine metabolic laboratory parameters—high-sensitivity C-reactive protein (hsCRP), hemoglobin A1C (HgbA1c), total cholesterol, triglyceride, high-density lipoprotein cholesterol (HDL-C), LDL-C, apolipoprotein A1 (ApoA1), and apolipoprotein B100 (ApoB100)—as well as muscle necroenzymes (creatine kinase (CK)), lactate dehydrogenase (LDH), aspartate aminotransferase (AST), and alanine aminotransferase (ALT) levels) were measured from fresh sera with Cobas c600 analysers (Roche Ltd., Mannheim, Germany) from the same vendor. The commercially available methods were used following the manufacturers’ protocols. All laboratory measurements took place at the Department of Laboratory Medicine, University of Debrecen, Debrecen, Hungary.

Serum concentrations of oxidized LDL (oxLDL) were detected by a commercially available solid-phase two-site enzyme immunoassay (ELISA) kit (Mercodia AB, Uppsala, Sweden). Measurements of oxLDL levels in the sera were performed according to the recommendations of the manufacturer. The intra- and inter-assay coefficients of variations were 5.5–7.3% and 4.0–6.2%, respectively, and the sensitivity was <1 mU/L.

Soluble intercellular adhesion molecule-1 (sICAM-1) and soluble vascular cell adhesion molecule 1 (sVCAM-1) levels were determined using a sandwich ELISA kit (R&D Systems Europe Ltd., Abingdon, UK). ELISA procedures were performed according to the manufacturer’s instructions. Intra- and inter-assay CVs ranged from 3.7% to 5.2% and 4.4% to 6.7% for sICAM-1 and 2.3% to 3.6% and 5.5% to 7.8% for sVCAM-1, respectively. Values were expressed in ng/mL.

### 2.3. Cardiovascular Disease Risk Assessment

CVD risk was estimated using the SCORE2 prediction model, carotid artery imaging, and the determination of carotid intima media thickness (IMT). SCORE2 is a standardized, validated risk assessment model used to estimate the 10-year risk of cardiovascular diseases [33]. It divides Europe into low-, medium-, and high-risk regions (Hungary is a high-risk area). Risk stratification assessment is based on gender, age, smoking status, systolic blood pressure, and total and HDL cholesterol levels. We used a SCORE2 calculator available online via the website of the European Society of Cardiology (ESC) [34]. Based on the SCORE2 values, the patients were classified into low- (<2.5%), medium- (2.5–7.5%), and high-risk (>7.5%) groups. If the 10-year risk is greater than 7.5%, lipid-lowering treatment is recommended according to international guidelines.

The identification of asymptomatic carotid plaques by Doppler ultrasound and IMT was determined at the Neurology Clinic of the University of Debrecen, Debrecen, Hungary. Measurement was performed using the Philips Epiq 5 (Philips International B.V., Amsterdam, The Netherlands) device with a 5–10 MHz transducer. In the first step, longitudinal and cross-sectional images were taken to rule out the presence of possible atherosclerotic plaques, as they distort the measurement of vessel wall thickness. If plaque was not visible, longitudinal images were taken 10 mm proximal to the division of the common carotid during end diastole, which were evaluated using the US Q-App IMT Release 9.0.x software (Philips International B.V.). The IMT is the distance between two echogenic bands—the lumen-intima border and the media adventitia border—which is given in millimetres. We performed 10 measurements on both sides, the average of which was calculated as the IMT value. The normal value of IMT is 0.40–0.80 mm. To score the results, we used a carotid plaque score system published by Ihle-Hansen et al. [35]: no plaque = 0 points, <50% stenosis = 1 point, 50–69% = 2 points, 70–100% = 3 points, plus 1 point if the plaque has a hypoechoic or ulcerated surface.

### 2.4. IIM Disease Activity and Muscular Side Effects of Lipid-Lowering Treatment

Myositis disease activity was evaluated based on IMACS core set measures using “Physician global activity”, “Patient global activity” 0–10 cm visual analogue scales (VAS), manual muscle test (MMT-8) scores, and CK levels. Muscle pain was also evaluated by the VAS scale [36,37].

Lipid-lowering therapy was administered after a shared decision with the individual patient. First, 10 mg/day of rosuvastatin was started in cases of medium and high CV risk patients (SCORE2 ≥ 7.5 and/or the presence of asymptomatic carotid plaque), in accordance with international recommendations. Furthermore, 10 mg of ezetimibe per day was recommended in cases of statin contraindication as follows: anti-HGGCR antibody positivity, previous statin-induced muscle pain or CK elevation, and actual significantly high CK level (3× above the normal upper limit). All patients who started the above-mentioned doses were naïve considering lipid-lowering therapy.

**Figure 1:**
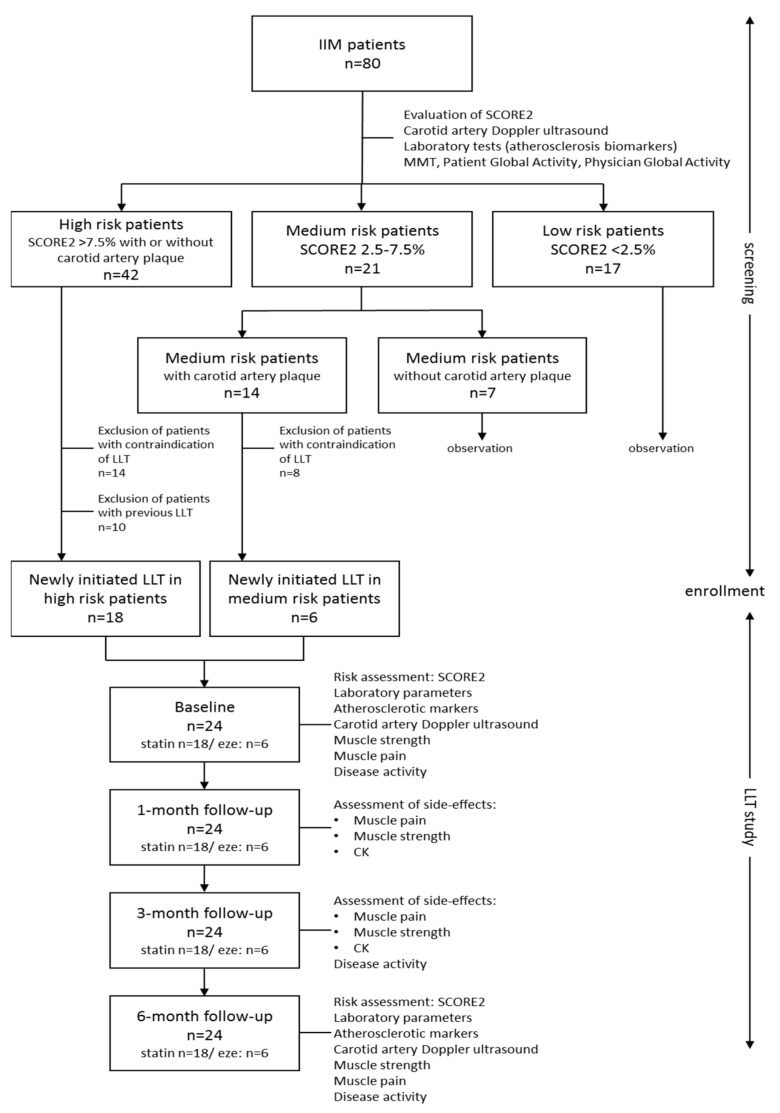
Study design flowchart.

All diagnostic and laboratory procedures were repeated in all cases after 1, 3, and 6 months independently of previous and actual lipid-lowering therapy. Carotid artery measurements were only tested at screening and 6 months after, as no significant change was expected in a short period of time. The study design is shown in Figure 1.

### 2.5. Statistical Analysis

Statistical calculations were performed using the Statistica 13.5.0.17 software (TIBCO Software Inc., Palo Alto, CA, USA). Graphs were made using GraphPad Prism 8.0 (GraphPad Software, San Diego, CA, USA). The normality of variables was checked using the Kolmogorov–Smirnov test. We performed a paired T-test for normally distributed variables and the Wilcoxon Matched Pairs test for skewed variables to detect the statistical differences after the 6-month follow-up. Data are presented as mean ± standard deviation or median (lower–upper quartiles). The statistical difference between categorical variables was analysed with the Chi-square test. A *p*-value ≤ 0.05 was considered statistically significant. Bonferroni-adjusted significance tests were applied to adjust *p*-values for multiple comparisons. When the calculated *p*-values were less than the Bonferroni threshold (*p* < 2.2 × 10^−3^), the difference was considered statistically significant.

## 3. Results

### 3.1. General Characteristics of the IIM Cohort

The general characteristics of the study cohort are summarized in Table 1. The mean age of IIM patients included in the study (n = 80) was 56.2 ± 13.4 years. The cohort is characterized by female dominance (67.5% women vs. 32.5% men). The median duration of myositis disease was 9 (5–15) years. Patients reported actual smoking in 17.5% (n = 14) of cases. Moreover, 49% (n = 39) of patients suffered from PM (including anti-synthetase syndrome and necrotizing myopathy) and 51% (n = 41) had DM. Furthermore, 60% (n = 48) of our patients had detectable myositis-specific (MSA) positivity and 43.7% (n = 32) had myositis-associated autoantibody (MAA) positivity. Anti-HMGCR antibody positivity was proven in only 2.5% (n = 2) of cases. The most common symptoms at IIM disease onset were muscle weakness (96.25%; n = 77), arthralgia/arthritis (81.25%; n = 65), and skin rashes (70%; n = 56). Internal organ involvement was reported less frequently: interstitial lung disease in 37.5% (n = 30) and dysphagia in 36.25% of cases (n = 29). Definitive myocarditis occurred in 16.25% (n = 13) of our patients. The leading traditional risk factors at screening were hypertension (71.3%) and diabetes mellitus (25%). The median disease activity was 1 (0–2.15) and the median CK value was 90.5 (62–185) IU/mL at screening, referring to low disease activity. The median, mean, standard deviation (SD), and quartile values of metabolic parameters, SCORE2, IMT, and biomarkers are presented in Table 2.

### 3.2. Patient Selection for Lipid Lowering Therapy (LLT)

**Figure 2:**
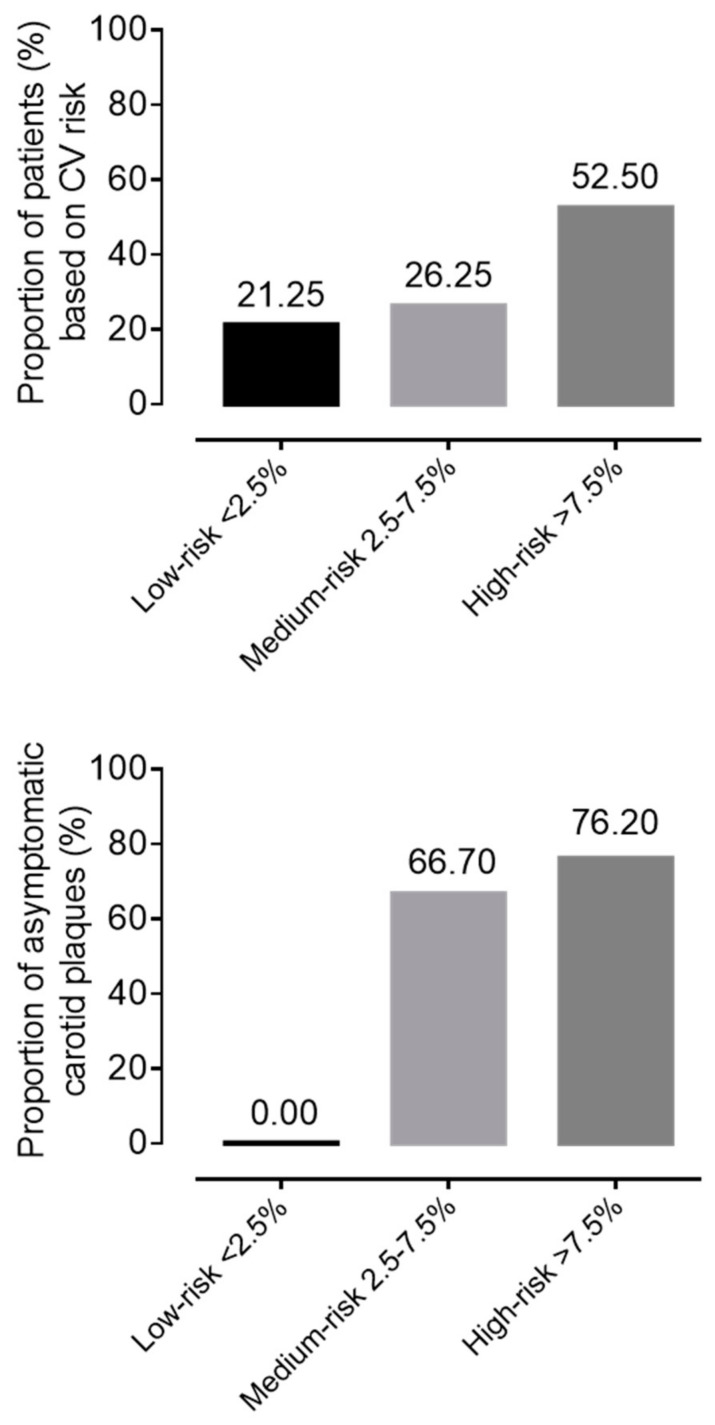
Proportion of patients with idiopathic inflammatory myopathy based on cardiovascular risk and asymptomatic plaque frequency.

During CV risk stratification, based on SCORE2, 21.25% (n = 17) of patients had low, 26.25% (n = 21) had medium, and 52.5% (n = 42) had high risk at screening. Evaluating the carotid artery ultrasound results, mean IMT values were in the normal range on both the right and left sides, but in 73.13% (n = 49) of patients, the carotid score was 1 point, which represents the presence of asymptomatic atherosclerotic plaques. Hyper echogenic, ulcerated, or pronounced plaques with more than 50% stenosis were detected in only 2.99% (n = 2) of subjects. In the low-SCORE2 risk group, we did not detect any carotid plaque. Meanwhile, 66.7% (14/21) of patients in the medium-risk group and 76.2% in the high-risk group had asymptomatic carotid artery plaques (Figure 2).

In the high-risk group, 10 patients were on continuous lipid-lowering therapy without any muscular side effects. We identified contraindication in 14 cases from the high-risk group and in 10 cases from the medium-risk group. We started 10 mg of rosuvastatin per day for 18 patients and 10 mg of ezetimibe per day for 6 patients. Adherence, patient-reported outcomes, and adverse effects were systematically recorded. All patients who started lipid-lowering treatment during this study were statin/ezetimibe-naive.

### 3.3. Efficacy of Lipid-Lowering Therapy

**Figure 3:**
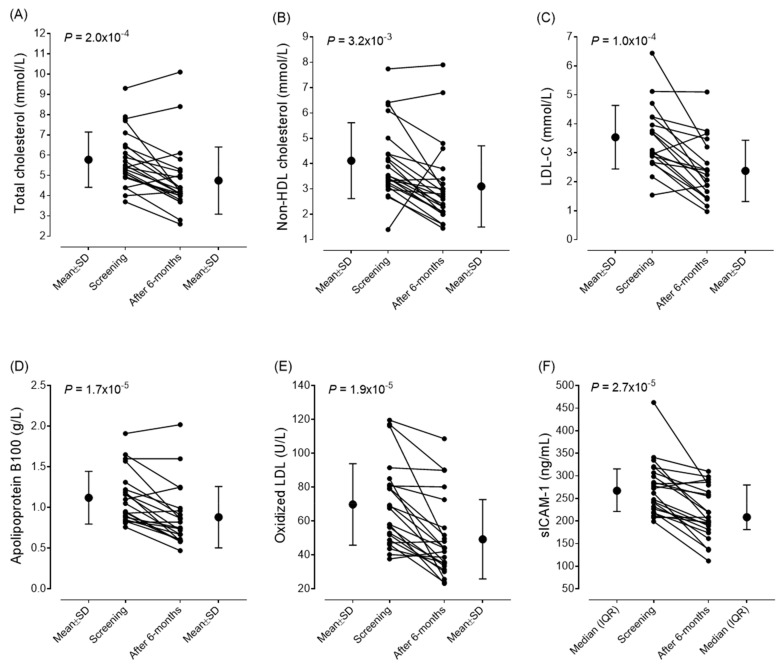
Individual changes in total cholesterol (**A**), non-HDL cholesterol (**B**), LDL-C (**C**), apolipoprotein B100 (**D**), oxidized LDL (**E**), and sICAM-1 (**F**) concentrations in patients with idiopathic inflammatory myopathy during the study period. Abbreviations: Apo B100, apolipoprotein B100; LDL-C, low-density lipoprotein cholesterol; non-HDL, non-high-density lipoprotein; oxLDL, oxidized LDL; sVCAM-1, soluble vascular cell adhesion molecule 1.

**Figure 4:**
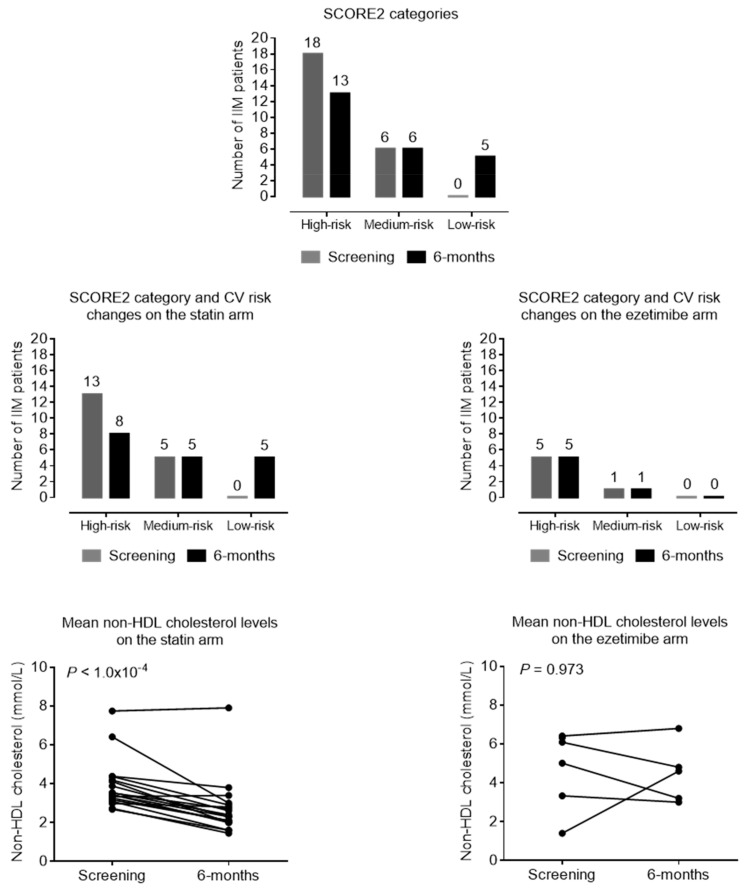
Proportion of patients in each CV risk category based on SCORE2 values and changes is CV risk based on SCORE2 values and non-HDL cholesterol level in statin and ezetimibe arms.

The changes in metabolic markers, atherosclerotic markers, and disease activity scores after six months of lipid-lowering treatment are summarized in Table 3. After the six-month follow-up, we observed a significant decrease in the mean values of total cholesterol (from 5.78 ± 1.36 to 4.75 ± 1.69 mmol/L; *p* < 0.001), non-HDL cholesterol (from 4.1 ± 1.49 to 3.1 ± 1.64 mmol/L; *p* < 0.001), and LDL-C levels (from 3.537 ± 1.095 to 2.377 ± 1.06 mmol/L; *p* < 0.001). We also found significant improvements in the values of oxLDL (from 69.83 ± 24.01 to 49.29 ± 23.39 U/L; *p* < 0.001), ApoB100 (from 1.12 ± 0.32 g/L to 0.88 ± 0.33 g/L; *p* < 0.001), and sICAM-1 (from 267.2 (217.9–311.35) to 208.7 (181.2–279.2) ng/mL; *p* < 0.001). In parallel, the SCORE2 scores also improved (from 9.75 ± 4.36 to 8.478 ± 3.58; *p* = 0.014). The mean IMT of carotid arteries did not show any changes during the observational period, and there was no progression in either thickness or plaque stage. The changes in vascular markers and CV scores are summarized in Table 3. The individual changes in lipid parameters, early markers of atherosclerosis, and sICAM-1 levels are demonstrated in Figure 3. Based on the SCORE2 values, 37.5% of the patients reached a lower risk category after treatment; thus, their cardiovascular risk was markedly reduced (Figure 4). This was based on decreased lipid levels; thus, other parameters of the SCORE2 system were not changed significantly.

Based on the SCORE2 improvements and non-HDL cholesterol level changes, there is a difference between ezetimibe and rosuvastatin in favor of statin, either in lipid lowering or risk reduction. In the statin arm, 56.6% of patients reached a lower risk category, while in the ezetimibe arm, the risk status did not change after half a year. Non-HDL cholesterol levels did not change in the ezetimibe arm (*p* = 0.973) but markedly decreased in the statin group (*p* < 0.001) (Figure 4).

### 3.4. Safety and Tolerability of Lipid-Lowering Therapy

During the follow-up period, no side effects of the lipid-lowering treatments were observed. The muscle pain VAS score determined by the patients (*p* = 0.463) and the CK levels (*p* = 0.212) did not change significantly. We did not observe any treatment-induced myopathy, myositis relapse, or progression during the follow-up period. No significant changes in muscle necroenzymes, disease activity, or muscle strength were detected. HgbA1c values did not indicate diabetogenic side effects of the statin treatment (Table 4).

## 4. Discussion

Based on our findings, it should be highlighted that due to carefully applied lipid-lowering treatment, the CVD risk of myositis patients was effectively reduced without any adverse muscular event. Lipid levels (total cholesterol and non-HDL cholesterol) and early atherosclerosis biomarkers (oxLDL, sICAM-1, and ApoB100 concentrations) significantly decreased, and there was no progression in IMT. Our results strengthen previous reports showing that statins are well tolerated in myositis patients with careful application [29,30].

The increased risk of accelerated atherosclerosis and cardiovascular diseases in systemic rheumatic diseases and IIM is well known. Several independent studies reported that in IIM, increased risk of cardiovascular mortality is related to the cardiac involvement of IIM and early atherosclerosis, mostly due to the traditional risk factors, the inflammatory milieu, and the applied corticosteroid therapy [2,3,4,5,6,7,8,9,10,11,12,13,14,26,27]. In patients with RMDs such as myositis, the European rheumatology guidelines [14] recommend a thorough assessment of traditional CVD risk factors. The use of cardiovascular prediction tools for the general population is recommended. Lipid management should also follow the recommendations used in the general population [14,33,38,39,40]. However, statin treatment in myositis patients has risks and special concerns, and no international guidelines have been established regarding the regularity of screening or risk-reduction strategies in these patients.

Based on our findings in cases of high CV risk and/or asymptomatic plaque, lipid-lowering treatment is recommended, where statin treatment is preferred in IIM patients without contraindication. Even though ezetimibe seems less effective based on our results, according to international guidelines, it could be used alone or in combination with lower statin doses, and in larger cohort studies, it effectively decreased CV risk [41,42]. The lower statistical efficacy of ezetimibe may be related to the previously proven modest reductions (approximately 20%) in LDL-C in addition to ezetimibe monotherapy [43].

Although the primary goal according to the guidelines is to reduce LDL-C levels, the risk of cardiovascular complications is more closely related to the serum level of ApoB100, as this parameter not only indicates the LDL particles but also the amount of highly atherogenic triglyceride-rich lipoproteins [41]. Indeed, reducing the non-HDL-C level is also important for the same reason. Therefore, a 21.4% reduction in ApoB100 and a 24.6% reduction in non-HDL-C levels indicate that the risk reduction is caused by the improvement of global dyslipidemia. The stabilization of atherosclerotic plaques, including the reduction in lipid content and intravascular inflammation within the plaque, is a further important outcome of lipid-lowering treatment [44,45]. However, measuring these parameters during routine clinical care is not currently feasible, and the IMT measurement we used is definitely not suitable for this purpose. Nevertheless, a favorable change in the sICAM-1 level, which characterizes endothelial function, may be an indirect indicator of plaque stabilization.

Since statins moderately increase the risk of developing newly onset type 2 diabetes mellitus [45], it is important to emphasize that after 6 months of treatment, the HbA1c value, which reflects carbohydrate metabolism, did not change significantly. Therefore, in IIM patients, the diabetogenic effect of statin treatment is likely to be less pronounced compared to the previously examined general patient population, in which the proportion of prediabetic and overweight patients may be higher.

In cases of contraindication of statin use, such as anti-HMGCR antibody positivity, disease activity, or previous statin-induced complaints, PCSK9 inhibitors or alternative therapies such as niacin or phytosterols could be chosen [13,38,39,44,45,46].

Our results also confirm that the CVD risk of IIM patients is high even after a longer disease duration. The high frequency of asymptomatic plaques suggests that screening using the carotid artery Doppler ultrasound is also recommended shortly after the diagnosis of IIM and regularly thereafter. As we detected high CVD risk in patients with an average disease duration of 9 (5–15) years, we suggest that it is necessary to repeat all the risk assessments regularly, not only in the first 5 years after IIM diagnosis but also later during the disease course. CVD risk assessment guidelines do not go into detail about what testing methods should be used and how frequently for risk assessment. They recommend the same CVD risk assessment as that in the international guidelines for the general population, which also does not include carotid artery Doppler testing [14,33,40].

Some possible limitations of the study must be mentioned. Due to the limited number of patients and the absence of a comparator group, the results need to be confirmed by further, large-scale, multicenter studies. The 6-month follow-up is certainly not long enough to confirm the beneficial effect of lipid-lowering treatment on plaque regression. The SCORE2 prediction model can only be safely used in Europe, as different ethnicities/races were not accounted for. Also, the calculated risks of SCORE2 are from the general population, and disease-related factors such as disease activity and steroid/immunosuppressive medication use are not considered. Therefore, we plan to continue the long-term follow-up of these patients. The measurement of further inflammatory and vascular biomarkers could contribute to exploring the favorable effects of lipid-lowering treatment. Nevertheless, this study provides important data supporting the effectiveness and safety of lipid-lowering treatment and underlines its role in cardiovascular risk reduction.

## 5. Conclusions

Based on our findings, we suggest that the cardiovascular risk of patients with myositis is high. As the treating physician—mostly the rheumatologist or neurologist—regularly follows IIM patients, it is necessary to advise or indicate CVD risk stratification in a timely manner and state recommendations for lipid-lowering therapy, considering the risks of adverse muscular events. Statin treatment can reach significant efficacy in risk reduction without side effects with care and follow-up. In case of statin infectivity, contraindication, or myotoxicity, clinicians have several different options, such as ezetimibe or PCSK9 inhibitors.

## Figures and Tables

**Table 1:** General characteristics of study population.

Characteristics of Study Population (n = 80)	
Mean age (years ± SD)	56.2 ± 13.4
Duration of disease (years)	9 (5–15)
Gender (female/male)	67.5% (n = 54)/32.5% (n = 26)
Smoking status (n)	17.5% (n = 14)
Polymyositis (including antisynthetase syndrome and necrotizing myopathy)	49% (n = 39)
Dermatomyositis	51% (n = 41)
Myositis-specific antibody (MSA) positivity	60% (n = 48)
Anti-Jo1 Antisynthetase	n = 18
Non-anti-Jo1 antisynthetase	n = 3
DM-specific antibodies (SAE, TIF1γ, NXP2, MDA5, Mi2)	n = 22
Anti-HMGCR antibody positivity	2.5% (n = 2)
Anti-SRP positivity	3.75% (n = 3)
Myositis-associated autoantibody (MAA) positivity (anti-Ro52/SSA, anti-Ku, anti-Pm/scl)	43.7% (n = 32)
Glucocorticoid treatment at screening	70% (n = 56)
**Clinical symptoms**	
Muscle weakness	96.25% (n = 77)
Skin rashes	70% (n = 56)
Interstitial lung disease	37.50% (n = 30)
Dysphagia	36.25% (n = 29)
Myocarditis	16.25% (n = 13)
Arthralgia	81.25% (n = 65)
Fever	17.50% (n = 14)
Raynaud’s phenomenon	38.75% (n = 31)
**Cardiovascular comorbidities**	
Hypertension	71.25% (n = 57)
Diabetes mellitus	25% (n = 20)
Heart failure	6.25% (n = 5)
Arrythmias	13.75% (n = 11)
Valvular problems	8.75% (n = 7)
Thromboembolic events	7.50% (n = 6)
Peripheral arterial diseases	17.50% (n = 14)
Stroke	0% (n = 0)
Acute myocardial infarction	3.75% (n = 3)

**Table 2:** Metabolic parameters, values of biomarkers of early atherosclerosis, and myositis disease activity markers at screening in patients with idiopathic inflammatory myopathy (n = 80).

Observed Parameters at Screening	Mean/Median	SD/Lower-Upper Quartiles
**Cardiovascular risk assessment:**		
Systolic BP (mmHg)	132.7	±15.341
Diastolic BP (mmHg)	81.85	±11.31
Total cholesterol (mmol/L)	5.718	±1.418
HDL-C (mmol/L)	1.566	±0.584
Non-HDL cholesterol (mmol/L)	4.175	±1.402
LDL-C (mmol/L)	3.438	±1.178
Triglyceride (mmol/L)	1.7	1.2–2.4
HgbA1c (%)	5.8	5.6–6.1
Score2 score	7.663	±5.178
**Assessment of atherosclerosis:**		
IMT right side average (mm)	0.591	±0.142
IMT left side average (mm)	0.632	±0.136
sVCAM-1 (ng/mL)	995.3	812.1–1191.6
sICAM-1 (ng/mL)	256.3	217.9–311.35
oxLDL (U/L)	71.049	±21.103
ApoB100 (g/L)	1.111	±0.307
ApoA1 (g/L)	1.649	±0.359
**Assessment of disease activity:**		
hsCRP (mg/L)	3.655	1.24–5.6
CK (IU/mL)	90.5	62–185
Patient’s global activity (0–10 cm scale)	2	0–3.5
Physician global activity (0–10 cm scale)	1	0–2.15
Manual muscle test score (0–150)	145	133.5–150

Abbreviations: ApoA1, apolipoprotein A1; ApoB100, apolipoprotein B100; BP, blood pressure; CK, creatine kinase; hsCRP, high sensitivity C-reactive protein; HDL-C, high-density lipoprotein cholesterol; HgbA1c, hemoglobin A1c; IMT, intima media thickness; LDL, low-density lipoprotein cholesterol; oxLDL, oxidized low-density lipoprotein; sICAM-1, soluble intercellular adhesion molecule-1; sVCAM-1, soluble vascular cell adhesion molecule 1.

**Table 3:** Changes in efficacy markers, metabolic markers, atherosclerotic markers, and SCORE2 values during the follow-up in patients with idiopathic inflammatory myopathy.

	Screening (n = 24)		6-Month Follow-Up (n = 24)		*p*-Values
	Mean/Median	SD/Lower-Upper Quartiles	Mean/Median	SD/Lower-Upper Quartiles	
**Lipid levels:**					
Total cholesterol (mmol/L)	5.78	±1.36	4.76	±1.69	<0.001 †
HDL-C (mmol/L)	1.66	±0.7	1.59	±0.56	0.452
non-HDL cholesterol (mmol/L)	4.11	±1.49	3.1	±1.64	0.003
LDL-C (mmol/L)	3.537	±1.095	2.377	±1.06	<0.001 †
Triglyceride (mmol/L)	1.515	1.2–2.4	1.535	0.94–2.13	0.976
**Assessment of atherosclerosis:**					
sVCAM-1 (ng/mL)	988.95	812.1–1191.6	1063.2	775.2–1236.2	0.465
sICAM-1 (ng/mL)	267.2	217.9–311.35	208.7	181.2–279.7	<0.001 †
oxLDL (U/L)	69.83	±24.01	49.29	±23.39	<0.001 †
ApoB100 (g/L)	1.12	±0.32	0.88	±0.39	<0.001 †
ApoA1 (g/L)	1.61	±0.29	1.68	±0.33	0.182
IMT right side average (mm)	0.599	±0.088	0.647	±0.123	0.079
IMT left side average (mm)	0.683	±0.124	0.653	±0.124	0.505
**Change in SCORE2: (primary endpoint)**					
Score2 point	9.75	±4.36	8.478	±3.58	0.014

Abbreviations: ApoA1, apolipoprotein A1; ApoB100, apolipoprotein B100; HDL-C, high-density lipoprotein cholesterol; IMT, intima media thickness; LDL-C, low-density lipoprotein cholesterol; oxLDL, oxidized low-density lipoprotein; sICAM-1, soluble intercellular adhesion molecule-1; sVCAM-1, soluble vascular cell adhesion molecule 1. † indicates that *p* passed the Bonferroni correction (*p* < 2.2 × 10^−3^).

**Table 4:** Changes in safety and tolerability markers, metabolic markers, and disease activity markers/scores during the follow-up in patients with idiopathic inflammatory myopathy.

	Screening (n = 24)		6-Month Follow-Up (n = 24)		*p*-Values
	Mean/Median	SD/Lower-Upper Quartiles	Mean/Median	SD/Lower-Upper Quartiles	
**Metabolic parameters**					
HgbA1c (%):	5.9	5.6–6.1	5.9	5.7–6.4	0.614
AST (U/L)	22	17.5–27.5	22	17–27	0.754
ALT (U/L)	20	10.55–30.5	25	17–31	0.126
GGT (U/L)	35.5	20–77	33	17–102	0.339
**Disease activity:**					
hsCRP (mg/L)	2.68	1.24–5.6	2.83	1.64–5.32	0.321
CK (IU/mL)	84	62–185	90.5	68–175	0.212
Patient’s global activity	2	0–2	0.5	0–2	0.197
Physician global Activity (0–10 cm)	0.75	0–1.15	1	0–1	0.594
Muscle pain VAS score (0–10 cm)	0	0–1.2	0	0–1	0.463
Manual muscle test score (0–150)	147	143–150	146	138–150	0.593

Abbreviations: ALT, alanine transaminase; AST, aspartate aminotransferase; CK, creatine kinase; GGT, glutamyl transpeptidase; HgbA1c, hemoglobin A1c; hsCRP, high sensitivity C-reactive protein; VAS, visual analogue scale.

## Data Availability

The data presented in this study are available on request from the corresponding author due to privacy.

## Abbreviations

ACR	American College of Rheumatology
anti–HMGCR	anti- 3-hydroxy-3-methylglutaryl coenzyme-A reductase antibody
ALT	alanine aminotransferase
ApoA1	apolipoprotein A1
ApoB100	apolipoprotein B100
ASS	antisynthetase syndrome
AST	aspartate aminotransferase
CV	cardiovascular
CVD	cardiovascular diseases
CK	creatine kinase
DM	dermatomyositis
ECG	electrocardiography
ELISA	enzyme linked immunoassay
ESC	European Society of Cardiology
EULAR	European Alliance of Associations for Reumatology
EZE	ezetimibe
GGT	glutamyl transpeptidase
HDL-C	high-density lipoprotein cholesterol
HgbA1c	hemoglobin A1C
HMG-CoA	3-hydroxy-3-methylglutaryl coenzyme-A reductase
hs CRP	high-sensitivity C-reactive protein
IBM	inclusion body myositis
IIM	Idiopathic inflammatory myopathies (IIM)
IMNM	immune-mediated necrotizing myopathy
IMT	carotid artery intima media thickness
LDH	lactate dehydrogenase
LDL-C	low-density lipoprotein-cholesterol
LLT	lipid-lowering therapy
MAA	myositis associated autoantibody
MMT	manual muscle test
MSA	myositis specific autoantibody
OM	overlap myositis
PCSK9	proprotein convertase subtilisin/kexin type 9
PM	polymyositis
RMD	rheumatic and musculoskeletal diseases
sICAM-1	soluble intercellular adhesion molecule-1
sVCAM-1	soluble vascular cell adhesion molecule 1
oxLDL	oxidized LDL
VAS	visual analogue scales
